# Insight of the Functional and Biological Activities of Coconut (*Cocos nucifera* L.) Protein by Proteomics Analysis and Protein-Based Bioinformatics

**DOI:** 10.3390/molecules27092987

**Published:** 2022-05-06

**Authors:** Jingrong Ma, Chuang Pan, Haiming Chen, Weijun Chen, Wenxue Chen, Ming Zhang, Qiuping Zhong

**Affiliations:** 1 College of Food Sciences & Engineering, Hainan University, 58 People Road, Haikou 570228, China; emma12pan06@outlook.com (J.M.); hnchwx@163.com (W.C.); zhangming-1223@163.com (M.Z.); hainufood88@163.com (Q.Z.); 2 Key Laboratory of Aquatic Product Processing, Ministry of Agriculutre and Rural Affairs, National R&D Center for Aquatic Product Processing, South China Sea Fisheries Research Institute, Chinese Academy of Fishery Sciences, Guangzhou 510300, China; silverpfoxc@hotmail.com; 3 Collaborative Innovation Center of Provincial and Ministerial Co-Construction for Marine Food Deep Processing, Dalian Polytechnic University, Dalian 116034, China

**Keywords:** coconut, shotgun proteomics, bioinformatics, globulins, antioxidant proteins

## Abstract

Coconut (*Cocos nucifera* L.) is one of the most critical economic crops in the tropics and sub-tropics. Although coconut protein has attracted more and more attention due to its nutritional potential, the lack of proteomic information has limited its practical application. The present study aimed to investigate the coconut meat proteome by shotgun proteomics and protein-based bioinformatic analysis. A grand total of 1686 proteins were identified by searching the National Center for Biotechnology Information (NCBI) protein database and self-constructed *C. nucifera* transcriptome repository. Among them, 17 and 9 proteins were identified as antioxidant proteins and globulins, respectively. Network analysis of the globulins referred to the sub-works of Cupin and Oleosin, and the antioxidant proteins were related to the sub-networks of glutathione metabolism and peroxisome. The bioactive peptides acquired by in-silico digestion of the targeted proteins have the potential to be applied as antioxidants and emulsifiers for both healthcare and food stabilization.

## 1. Introduction

A global focus on sustainability has accelerated research into alternative non-animal food protein sources and functional food ingredients [1]. Proteins sourced from plants are considered valuable ingredients by the food industry in preparing functional foods [2]. Coconut (*Cocos nucifera* L.) belongs to the Palm family (Arecaceae) with a 2n = 32 ploidy and is one of the most critical economic crops in the tropics and sub-tropics [3]. According to the Food and Agriculture Organization, the annual world production of coconut is approximately 60.7 million tons [4]. The protein content of fresh coconut meat is about 2.6–4.4% on a wet basis (*w*/*w*), and the large mass of coconut grown globally makes recovery of these proteins desirable [5]. Most of the studies on coconut meat mainly focus on the effects of processing on the nutritional quality of coconut proteins [6] and characterizing the major proteins present in coconut via classical biochemical purification tools [7,8]. Although coconut is a large part of the human diet with potential health benefits [9], information on the components, functional, and biological activities of coconut proteins are scarce. Therefore, evaluating the protein components in coconut and understanding its functional and biological activities are essential, which is good for the processing and application of coconut.

Proteomics is being increasingly used to assess the diversity, quality, and bioactivity of plant proteins, such as almond kernel [10], lupin seed [11], maize seed [12], avocado seed [13], and soybean [14]. It can describe the composition, function, interaction, and modification of direct and coordinated cell activities over a given time or under specific environmental cell conditions of proteins [3]. Liquid chromatography-tandem mass spectrometry (LC-MS/MS) is the method of choice for shotgun proteomics [15]; based on this method, the proteomics analysis could acquire a large-scale protein identification. Proteomics analysis has been proved much better than two-dimensional electrophoresis (2-DE), which has certain disadvantages, such as the limited number of identified proteins, narrow linear detection dynamic range, long analysis time, and low sensitivity and reproducibility [15]. At present, proteomics research in coconut meat is poorly represented. To our knowledge, few studies have been reported. D’Amato, Fasoli, and Righetti [16] analyzed the proteome of coconut milk and identified 307 unique gene products; the most abundant proteins were the glutelin and 7S globulin, homologous to proteins present in oil palm and that have a nutrient reservoir activity. Huang et al. [17] investigated the proteome of coconut meat and found 200 protein spots on 2-DE maps; the identified proteins were limited, which were nonetheless very informative in that some of the proteins in coconut endosperm were found to be “storage proteins”. Recently, coconut storage proteins have been paid growing attention to their nutritional and health benefits [3]. Conclusively, protein-based bioinformatic information on the coconut is relatively scarce, and further studies are needed.

The lack of information regarding the functional and biological properties of individual protein or protein groups of coconut has limited their applications. Given these circumstances, the present study aimed to identify and characterize the coconut meat proteins by a shotgun proteomic approach. Meanwhile, the bioinformatics analysis was performed to illuminate the functional and biological activities of all the identified proteins. Furthermore, different protein-based bioinformatic analyses were combined to acquire the bioactive peptides of globulins and antioxidant proteins and reveal their potential function.

## 2. Results and Discussion

### 2.1. Coconut Meat Proteome

A shotgun proteomics analysis was carried out to obtain comprehensive proteome information on coconut meat. In the NCBI protein database, a total of 22,572 proteins for *C. nucifera* were registered. In addition, a transcriptome repository of *C. nucifera* was constructed by de novo assembly as a supplementary database.

**Figure 1:**
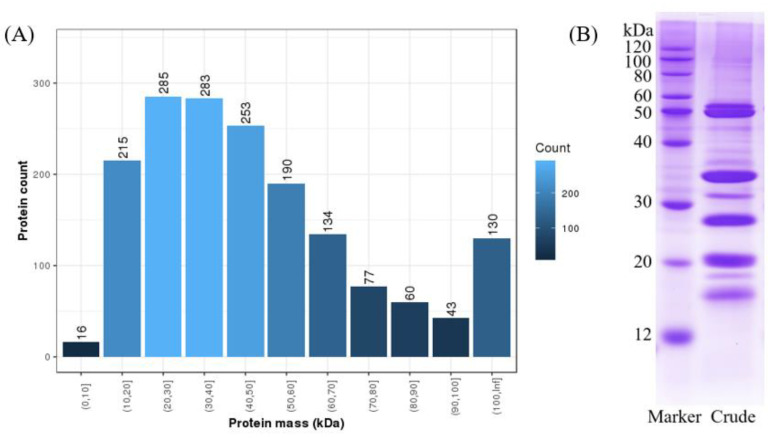
(**A**) Distribution of protein mass. (**B**) SDS-PAGE 12% profiles of the extracted proteins of *C. nucifera* sample.

The preliminary analysis of the coconut meat proteome showed that the proteins in coconut are abundant. A total of 279,953 spectra were obtained from the sample, 16,199 spectra were matched, and 1686 proteins were identified by searching in both the NCBI protein database and transcriptome repository, which were much more than the proteins identified by previous studies [16,17]. The molecular weights of the proteins were predominantly distributed among 10 and 60 kDa: a total of 1226 proteins were found in this range (72.72%) (Figure 1A). In addition, the SDS-PAGE analysis corresponds well with the identification results (Figure 1B). Demason and Sekhar [18] reported that reduced coconut proteins fractionated into seven major bands ranging from 17 to 55 kDa. Kwon, Park, and Rhee [19] also found that coconut proteins consisted of about seven major bands with molecular weights of between 14 and 52 kDa in a reduced state. These previous results are consistent with the protein pattern in our study.

### 2.2. Functional and Bioinformatic Analysis

**Figure 2:**
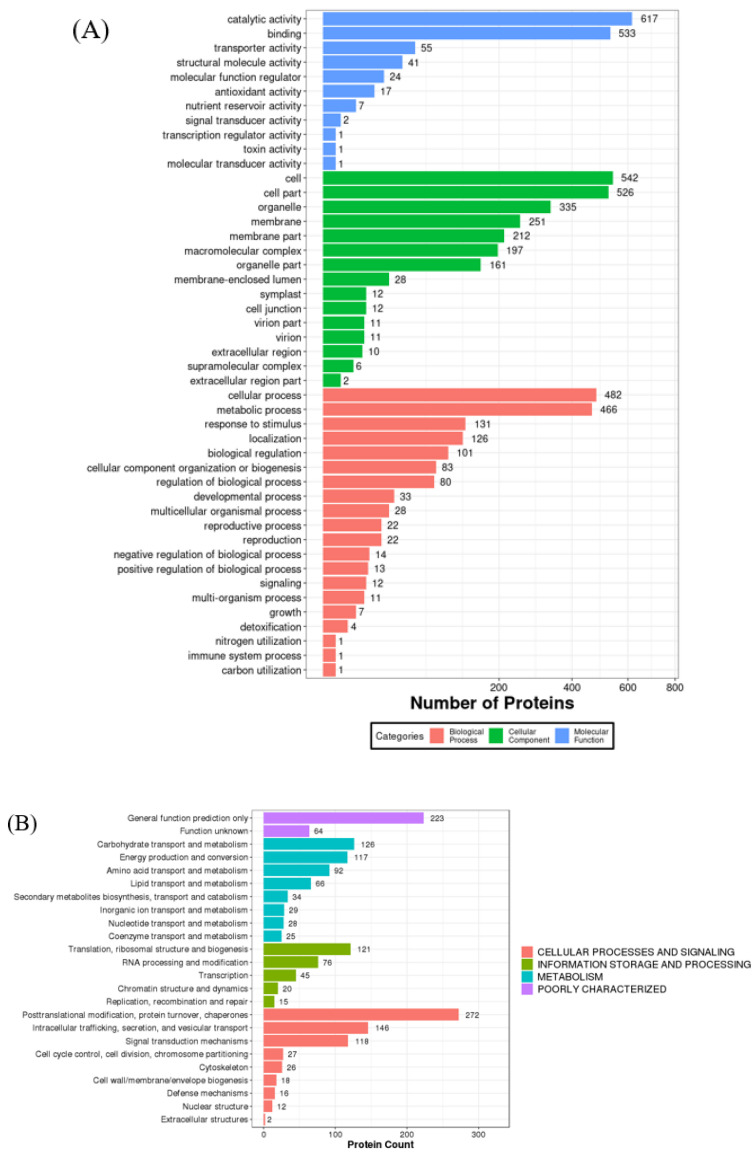
(**A**) Gene ontology (GO) term enrichment for coconut derived proteins. (**B**) Bar chart of the Eukaryotic orthologous group (KOG) analysis.

As the functional agents which are created by genomic transcripts and are the final products of gene expression, proteins function through an integral and coordinated network to regulate metabolism in cells, tissues, and organisms as a whole [3]. The functional classification and bioinformatic analysis of all the identified proteins were based on the molecular function, biological process, and cellular component (Figure 2).

#### 2.2.1. Molecular Functions

As shown in Figure 2A, the results of molecular functional analyses demonstrated that the proteins with catalytic (36.6%) and binding (31.6%) activities were predominant in these groups. The exuberant metabolism and biosynthesis in the coconut endosperm need various proteins with different catalytic activities. Therefore, proteins represent the catalytic activities in various aspects, such as hydrolase activity, transferase activity, and phosphatase activity. Multiple studies have analyzed the individual enzymatic functions of coconut proteins during seed development, such as the tyrosine kinase activity [20,21], polyphenol oxidase activity [22], and lipase activity [23]. For fruit development, coconut meat is imbued with various nutrients, such as minerals and bioactive molecules, so a mass of proteins with binding abilities is needed.

Seed storage proteins have the pivotal role of providing the seedling with amino acids and nitrogen source for metabolism during germination and growth [24]. In this study, seven proteins were identified with nutrient reservoir activity, and all the proteins are globulins except the glutelin (ID: KAG1339186.1). Globulin is the predominant protein in coconut meat (40%) and the essential storage protein as well [25]. In recent years, there are many studies on its purification and identification [7,8]. 11S globulin (300–400 kDa, also known as cocosin), owning six subunits (50–60 kDa), accounted for 86% of the coconut globulin, whereas 7S globulin (150–200 kDa, also called vicilin-like protein), owning three subunits (40–70 kDa), only take up 14%. In this study, six 7S globulins (ALQ56981.1, CL8941.Contig1, CL3433.Contig1, CL4719.Contig2, CL4719.Contig3.1, and KAG1368674.1) and three 11S globulins (KAG1361520.1, ASQ40963.1, and Unigene39216) were identified (Table 1). This result could guide the extraction and purification of coconut globulin. Globulin has many potential applications in the food industry because of its good functional activity, such as emulsifying properties due to its amphiphilic molecular structure [26]. Currently, the most widely used protein-based emulsifiers are dairy proteins, like casein and whey proteins. However, animal protein presents growing costs and limited supply. It has been highly associated with climate change, freshwater depletion, biodiversity loss, and hazards for human health related to cardiovascular diseases and others [27]. Plant proteins are increasingly being used as a versatile alternative, replacing animal sources and functional ingredients for product formulation. The big part of globulin of coconut may have good application in emulsifiers.

Hitherto, the bioactive properties of coconut proteins are rarely investigated. Li et al. [28] found that the coconut proteins exhibited radical-scavenging activity and ion chelating ability; they can also protect DNA from oxidative damage. In this work, 17 proteins were identified with antioxidant activities (Table 2). Most identified antioxidant proteins were peroxidase, superoxide dismutase, catalase isozyme, and glutathione reductase. The protein mass of the antioxidant proteins is mainly concentrated in low molecular weight (18–35 kDa). Environmental or biotic stress could generate reactive oxygen species (ROS) and break the cellular redox balance. Therefore, a large variety of antioxidant enzymes are needed. Antioxidants play a vital role in both food systems and the human body to reduce oxidative stress. Since synthetic antioxidants have been suspected of threatening human health, antioxidants from natural sources have attracted more attention [28]. The coconut proteins with antioxidant activities identified here could provide a reference for their further applications.

#### 2.2.2. Cellular Components

Regarding cellular components, the coconut proteins were mainly distributed in the cell, cell part, organelle, membrane, membrane part, macromolecular complex, and organelle part (Figure 2A). The proteins are present in coconut meat in various forms and cellular locations, such as as a component of the cell wall. They also bind to other cellular components, such as carbohydrates and membranes. Lipids are the primary source of energy in coconut endosperm, thus a great number of membrane structure is needed for its storage. Generally, membrane proteins possess emulsifying properties due to their amphiphilic structure. Yesiltas et al. [1] found that the proteins which are membrane-associated or form highly complex membrane-like macrostructures are more likely to include highly hydrophilic–hydrophobic regions. Therefore, except for the globulin, the membrane proteins also have potential applications as emulsifiers.

#### 2.2.3. Biological Processes

As shown in Figure 2A, the proteins primarily participate in the cellular process, metabolic process, response to stimulus, localization, and biological regulation when focusing on the biological processes. These results indicated that, as a vegetative organ, coconut meat needs to perform various biological processes, and most coconut proteins are involved in multiple types of metabolism and cellular processes. From the KOG analysis (Figure 2B), we know that the main metabolic processes are carbohydrate transport, energy production, amino acid transport, and lipid transport, which correspond to the properties of seed proteins for embryo development. The ability of coconut to resist highly stressed environments originates from biological regulation and responses to stimulus behavior—most antioxidant proteins respond to oxidative stress and cell redox homeostasis. In addition, the heat shock proteins (HSPs) can assist protein folding and help to refold damaged proteins under stressful conditions [29]. This contributes to a better understanding of the molecular mechanisms of tropical plants responding and adapting to environmental change and facilitates the development of some anti-stress proteins. Additionally, further investigations on the functions of poorly known proteins are needed, considering there are 223 proteins that were poorly characterized and 64 proteins were uncharacterized (Figure 2B).

The globulins and antioxidant proteins from coconut meat may be promising in future applications as emulsifiers and antioxidants, respectively; more knowledge about them is needed. Therefore, in this work, we chose globulins and antioxidant proteins for the further bioinformatic analysis (network analysis and putative bioactive peptides).

### 2.3. Network Analysis for Globulins and Antioxidant Proteins

**Figure 3:**
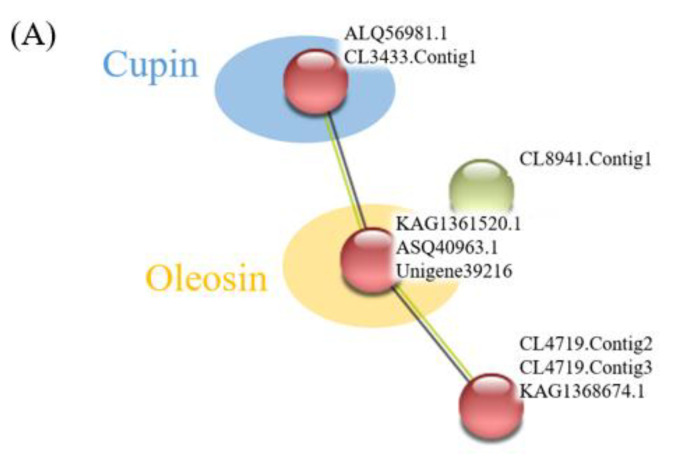

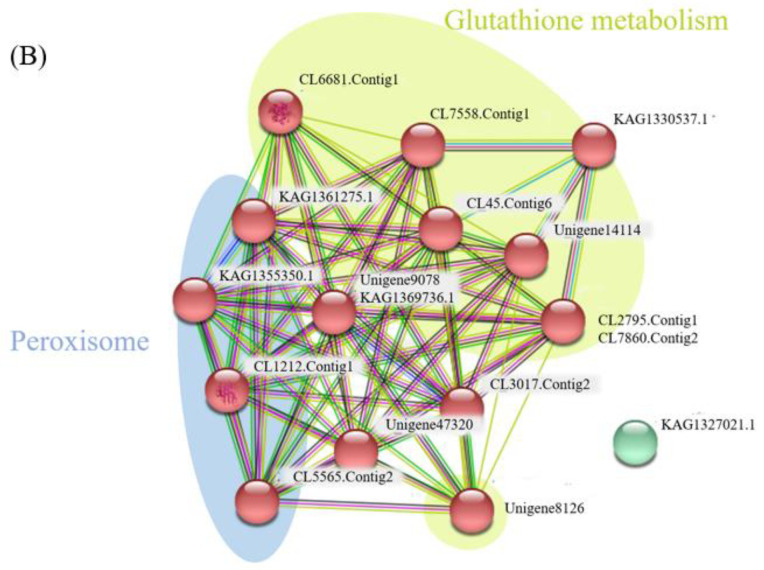
Protein network for the globulin (**A**) and antioxidant proteins (**B**) using the STRING (v11.0) software. The solid lines represent the physical direct interactions.

Proteins do not exist independently in vivo, as their functions are associated with, or regulated by, other proteins [30]. Therefore, we performed a network analysis of the target proteins using protein–protein interaction. All the globulins and antioxidant proteins identified from the coconut meat proteome were merged on the STRING software (v.11.0, https://string-db.org/, accessed on 21 September 2021) to obtain cross-correlation information. The oil palm (*Elaeis guineensis* Jacq), date palm (*Phoenix dactylifera* L.), carnauba palm (*Copernicia prunifera* (Mill.) H.E.Moore), and coconut palm (*Cocos nucifera* L.) all belong to palm family that is restricted to tropical and subtropical climates [31]. The *P. dactylifera* L. was selected as a reference organism considering the fact that the genome of *C. nucifera* is not available in the STRING database. As shown in Figure 3, there were 4 nodes (9 proteins) and 2 edges (interactions) for the globulins (Figure 3A), and 15 nodes (17 proteins) and 78 edges (interactions) for the antioxidant proteins (Figure 3B).

The storage proteins are a group of plant structurally-conserved polypeptides lacking catalytic activities. Along with the seed development, they accumulate in the endosperm and catabolize to be used as a nitrogen source to support the growth of the seedling [3]. The topological analysis of the globulins revealed two sub-networks (Figure 3A): Cupins with two vicilin-like proteins (ALQ56981.1 and CL3433.Contig1.) and Oleosin with three 11S globulins (KAG1361520.1, ASQ40963.1, and Unigene39216). Cupins are classified as storage proteins and are involved in such other relevant functions as seed germination and stress defense [24]. The results suggested that the two vicilin-like seed storage proteins (ALQ56981.1 and CL3433.Contig1.) are mainly involved in seed development and external pressure alleviation. The lipids are major nutrient reserves in the Arecaceae; the 11S globulin, as the Oleosin, further proves it is the leading seed storage protein related to the endosperm germination progression, complexed with lipids. Similarly, Nascimento et al. [31] found the 11S globulin seed storage proteins in the endosperm of *Euterpe oleracea* decreased dramatically with germination progression.

The topological analysis of the antioxidant protein also revealed two sub-networks (Figure 3B): glutathione metabolism with eight proteins (CL6681.Contig1, Unigene8126, CL7558.Contig1, KAG1330537.1, CL45.Contig6, Unigene14114, CL2795.Contig1 and CL7860.Contig2.) and peroxisome with four proteins (KAG1361275.1, KAG1355350.1, CL1212.Contig1 and CL5565.Contig2.) Most antioxidant proteins from the glutathione metabolism sub-networks were mainly involved in cellular processes and signaling, such as posttranslational modification, protein turnover, and chaperones. The proteins in the peroxisome sub-network are designated with metabolism, including inorganic ion transport and metabolism.

### 2.4. Putative Bioactive Peptides for Globulins and Antioxidant Proteins

Food protein-derived peptides are analyzed using different approaches, including in silico, in vitro, and ex vivo/in vivo studies [32]. The in-silico digestion (known as bioinformatics) is based on the elaboration of databases of peptides by computer technologies; it applies to information technologies for studying the potential of proteins as sources of peptides. Considering the advantages of in-silico digestion, studying bioactive peptides, is less costly and time-consuming. Therefore, the final part of the work is dedicated to investigating the possible physiological roles of peptides deriving from globulins and antioxidant proteins—the major storage proteins and bioactive proteins in coconut meat. They were subjected to in-silico digestion by sequential hydrolysis with pepsin and trypsin.

For the globulins, after digesting by pepsin, a total of 11 peptides were selected as potential bioactive peptides (Table 3) with a PeptideRanker score higher than 0.8 (7–27 amino acid residues), all of which corresponded to seven proteins. After tryptic digestion, 50 tryptic peptides (7–34 amino acid residues) were predicted as bioactive peptides (Table 4). The potential bioactive peptides obtained from all the globulins showed as non-toxic. For the antioxidant proteins, digestion by pepsin released 17 potential bioactive peptides (Table 5), which corresponded to eight proteins. Most of the bioactive pepsin digested peptides showed as non-toxic except for the APPVCCRF obtained from CL2795.Contig1_coconut and the FHPPMVSF which was obtained from KAG1369736. After tryptic digestion, 46 peptides (7–34 amino acid residues) were considered as bioactive peptides (Table 6). Similarly, most of the bioactive trypsin digested peptides showed non-toxic except the GVSFPFPVSSSSAAPPVCCR obtained from protein CL2795.Contig1_coconut. The toxicity of the bioactive peptides would impede their application in the food industry. It was previously indicated that Val, Thr, Arg, Gln, Met, Leu, Lys, Ile, Phe, and Ala are primary components of the non-toxic antioxidant peptides, while the Pro, His, Cys, and Asn amino acid residues are predominant in toxic peptides [33], which agreed well with the present results. In addition, the bioactive peptides obtained after digestion by pepsin were far less than that obtained by trypsin, however, oligopeptides were dominant after pepsin digestion.

Globulin is the main protein in coconut meat, but research on its bioactive peptides is scarce. Li et al. [28] demonstrated that the globulin had relatively high antioxidation properties, and the peptides after tryptic cleavage showed antioxidant activity. Although the coconut globulin lacks catalytic activities, the functional peptides released after digestion by trypsin and pepsin in this work could provide preliminary data for future studies and applications. For example, emulsifying peptides represent a class of promising biomolecules to replace chemical emulsifiers in food emulsions. Amphiphilicity is crucial in peptide self-assembly, and therefore also of tremendous importance in the interfacial properties of peptides [1]. The results suggest that the QSFQQSESEQQGEKGQRRRSRDEHQRI from KAG1361520.1 and most peptides digested by trypsin, determined in this study, with both positively and negatively charged polar amino acids, may be used as emulsifiers.

In recent years, an increasing number of studies have been conducted on the antioxidant capacity of plant protein-derived peptides. Plant proteins have been considered as a green source of antioxidant peptides, which help save energy, strengthen the treatment of oxidation-related diseases, and delay the oxidation of food [34]. Proteins and peptides are good antioxidants due to their ability to inhibit lipid peroxidation in the human body. Their mechanisms of action include inactivating ROS, scavenging free radicals, chelating pro-oxidative transition metals, and reducing hydroperoxide formation [35]. Plants continuously generate ROS under various abiotic and biotic stress conditions, such as heat, drought, high salinity, cold, and pathogen infection. The ascorbate (AsA)-glutathione (GSH) cycle plays an important role in the detoxification of ROS, but only AsA is specific and highly abundant in plants [36]. Thus, the potential digested peptides from the antioxidant protein of the glutathione metabolism sub-networks determined in this study may be utilized as natural antioxidants for healthcare. These potential bioactive peptides need to be synthesized and further verified. Nevertheless, compared with classical approaches, bioinformatic analysis is a faster and cheaper alternative method that reduces the number of potential targets to be researched.

## 3. Materials and Methods

### 3.1. Materials

The fresh local Hainan Tall coconut (*C. nucifera*) specimens were harvested in Wenchang, Hainan, China. Coconut meats were collected and immediately frozen in liquid nitrogen before being transported to the laboratory, where they were stored at −80 °C before use.

### 3.2. Transcriptome De Novo Assembly

The transcriptome repository was assembled according to the method reported by Pan et al. [36]. The polyA-tailed mRNA was enriched and fragmented before the synthesis of double-strand DNA and adaptor ligation. The PCR products were then heat-separated to a single strand of DNA and circularized with a bridge primer to obtain the DNA library before sequencing. The raw data were filtered to obtain the clean reads performed with Trimmomatic (v0.36). De novo assembly of the clean reads was performed with Trinity (v2.0.6). Quality of de novo assembly was analyzed via BUSCO (v5.0.0) (http://busco.ezlab.org/, accessed on 8 April 2021) [37].

### 3.3. Protein Extraction

Fresh coconut meat (300 mg) was grounded with homogeneous buffer containing 20 mM Tris-HCl (pH 8), 30% saccharose, 2% β-mercaptoethanol, 1 mM dithiothreitol (DTT), 100 mM EDTA, 1% Triton X-100 (Sigma-Aldrich, St. Louis, MA, USA), and protease inhibitor. This mixture was further blended with 2 volumes of saturation phenol, which was centrifuged at 25,000× *g* for 15 min at 4 °C after being shaken for 15 min. The supernatant was collected afterward and mixed with 5 volumes of cold methyl alcohol and 10 mM DTT, followed by incubation at −20 °C for 2 h first and centrifugation at 25,000× *g* for 15 min at 4 °C. The acquired sediments were mixed with cold acetone (1 mL) and incubated at −20 °C for 30 min. The protein precipitate was collected after air-drying the sediments from centrifugation. To reduce the protein, the protein precipitate was re-dissolved by the lysis buffer containing 20 mM Tris-HCl (pH 8), 7 M urea, 2 M thiourea, 4% SDS, 2 mM EDTA, and protease inhibitor. The reduction was carried out by adding DTT to a final concentration of 10 mM in a 56 °C water bath for 1 h. Then, 55 mM iodoacetamide (IAM) was added, and the solution was placed in a dark room. After 45 min, cold acetone (1 mL) was added to the tube, followed by incubation at −20 °C for 2 h, centrifugation, and air-drying as above, to obtain the protein precipitate, which was grounded with lysis buffer (without SDS) and centrifuged again. The protein concentration in the supernatant was determined by the Bradford method [38].

### 3.4. SDS-Polyacrylamide Gel Electrophoresis

SDS-polyacrylamide gel electrophoresis (SDS-PAGE) was performed with a 12% (*w*/*v*) acrylamide resolving gel, as previously described by Laemmli [39]. Proteins (10 μg) were loaded on the gel. Pre-stained Color Protein Marker (12–120 kDa, Beijing Solarbio Science & Technology Co., Ltd., Beijing, China) was used as a molecular weight indicator. Electrophoresis was conducted in a vertical electrophoresis unit (Bio-Rad Mini-PROTEAN^®^Tetra, Bio-Rad Laboratories, Hercules, CA, USA) at 80 V for 30 min, followed by 120 V for 60 min. The gel was then stained with Coomassie blue R-250 stain solution (45% methanol, 10% acetic acid, and 0.25% R-250), decolorized with water/methanol/acetic acid (5:4:1, *v*/*v*), and photographed.

### 3.5. In-Solution Protein Digestion

A total of 150 μg of coconut protein was denatured with 8 M urea and then reduced with 5 mM Tris (2-carboxyethyl) phosphine (TCEP) at 37 °C for 60 min. After alkylating (40 min, in the dark) with 55 mM IAM in 50 mM ammonium bicarbonate, the urea concentration was decreased by diluting with 50 mM ammonium bicarbonate. Proteins were digested with trypsin (1:20 protease-to-protein ratio) at 37 °C for 4 h.

### 3.6. Proteomic Identification

The digested peptides were dissolved with mobile phase A (2% acetonitrile, 0.1% formic acid). The supernatant was collected by centrifuging (20,000× *g*, 10 min) and analyzed by LC-MS/MS. The sample (2 μg) was first enriched in a trap column and desalted, and then entered a self-packed C_18_ column (75μm internal diameter, 3 μm particles, 25 cm column length) and was separated at a flow rate of 300 nL/min by a 60 min linear gradient from 5% to 80% solution B (98% acetonitrile, 0.1% formic acid). The peptides that were separated by liquid-phase chromatography were ionized by a nanoESI source and then passed through a tandem mass spectrometer Q-Exactive HF X (Thermo Fisher Scientific, San Jose, CA, USA) for DDA (Data Dependent Acquisition) mode detection. All MS/MS spectra were analyzed by the Mascot (v2.3.02) search engine against the National Center for Biotechnology Information (NCBI) protein database and the transcriptome de novo database constructed. The Percolator algorithm was used to filter the results by keeping a false discovery rate (FDR) below 1% [40].

### 3.7. Bioinformatic Analysis

The non-redundant protein IDs were submitted to the PANTHER program (https://geneontology.org/, accessed on 19 May 2021) for gene ontology (GO) enrichment analysis. The classification is based on protein class and biological processes. The identified proteins were blasted in the eukaryotic orthologous groups (KOG) database for the KOG annotation. Network analysis for globulins and antioxidant proteins was implemented by submitting the selected proteins dataset to the STRING software (v11.0, https://string-db.org/, accessed on 21 September 2021). Cluster networks were created using the MCL inflation algorithm, and a value of 3 was selected for all analyses [41].

### 3.8. Bioactive Peptides Prediction

Bioactive peptides encrypted in the globulins and antioxidant proteins were predicted by in-silico protein hydrolysates with pepsin and trypsin enzymes. All the proteolytic digestions were performed in-silico using the MS-Digest software in ProteinProspector (v.5.24.0, http://prospector.ucsf.edu/prospector/mshome.htm, accessed on 21 September 2021). To evaluate the results, the PeptideRanker (https://distilldeep.ucd.ie/PeptideRanker/, accessed on 21 September 2021) was used to rank the potential peptides at a threshold of 0. In addition, for ensuring the application of the activated peptides, they were put into ToxinPred (https://webs.iiitd.edu.in/raghava/toxinpred/index.html, accessed on 21 September 2021) for toxicity identification.

## 4. Conclusions

For such an important agricultural species, coconut has received only limited study. In the present study, the coconut meat proteome was researched based on shotgun proteomics and protein-based bioinformatic analysis. The proteins in coconut are abundant with a grand total of 1686 proteins identified. In addition, a total of 17 proteins were identified as antioxidant proteins and 9 globulins were found through the functional and bioinformatic analysis. The globulins and antioxidant proteins and their corresponding bioactive peptides may be promising in future applications as emulsifiers and antioxidants. This work presents a systematic analysis of coconut meat proteins and could direct the development of coconut processing technology. The bioinformatic analysis sometimes cannot faithfully reflect the functional and biological activities because it misses out on several important aspects, such as actual environment variations and biotic conditions effects. However, compared with classical approaches, bioinformatic analysis is a broader, faster, and deeper alternative method for a system searching for uncharacterized proteins; it is a feasible way to make predictions and will have a greater application in future foodomics.

## Figures and Tables

**Table 1:** *C. nucifera* proteome (globulin, FDR < 1%).

N	Protein_ID	Protein Mass (kDa)	Description	Uni. Pep.	Cov. (%)
1	ALQ56981.1	54.81	vicilin-like protein (*Cocos nucifera*)	2	3.47
2	CL8941.Contig1_coconut	90.00	vicilin-like seed storage protein At2g18540 (*Elaeis guineensis*)	5	5.12
3	KAG1361520.1	52.71	cocosin 1 (*Cocos nucifera*)	15	28.60
4	ASQ40963.1	52.62	cocosin (*Cocos nucifera*)	12	50.43
5	CL3433.Contig1_coconut	52.48	vicilin-like seed storage protein At2g28490 (*Elaeis guineensis*)	8	12.16
6	Unigene39216_coconut	51.48	cocosin 1 (*Elaeis guineensis*)	32	55.97
7	CL4719.Contig2_coconut	64.75	7S globulin (*Elaeis guineensis*)	11	41.83
8	CL4719.Contig3_coconut	65.98	7S globulin (*Elaeis guineensis*)	19	34.32
9	KAG1368674.1	50.38	putative vicilin-like antimicrobial peptides 2-1 (*Cocos nucifera*)	4	26.37

N: Identification Numbers, FDR: False Discovery Rate, Uni. Pep.: Unique Peptides, Cov.: Protein coverage.

**Table 2:** *C. nucifera* proteome (GO class=antioxidant activity, FDR < 1%).

N	Protein_ID	Protein Mass (kDa)	Description	Uni. Pep.	Cov. (%)
1	CL7558.Contig1_coconut	26.66	phospholipid hydroperoxide glutathione peroxidase 1, chloroplastic (*Cocos nucifera*)	2	6.58
2	CL2795.Contig1_coconut	28.12	probable phospholipid hydroperoxide glutathione peroxidase (*Elaeis guineensis*)	3	12.55
3	Unigene9078_coconut	22.16	peroxiredoxin-2 (*Capsaspora owczarzaki* ATCC 30864)	1	5.03
4	CL7860.Contig2_coconut	25.27	probable glutathione peroxidase 5 (*Elaeis guineensis*)	3	10.71
5	Unigene47320_coconut	18.82	peroxiredoxin-2C (*Elaeis guineensis*)	1	8.05
6	CL1212.Contig1_coconut	19.83	superoxide dismutase [Cu-Zn], chloroplastic (*Elaeis guineensis*)	2	14.67
7	Unigene8126_coconut	33.93	probable L-ascorbate peroxidase 4, peroxisomal (*Elaeis guineensis*)	3	7.77
8	Unigene14114_coconut	22.19	probable phospholipid hydroperoxide glutathione peroxidase isoform X1 (*Elaeis guineensis*)	2	8.67
9	KAG1327021.1	36.53	peroxidase 63 (*Cocos nucifera*)	3	9.52
10	KAG1355350.1	106.60	putative Catalase isozyme 1 (*Cocos nucifera*)	4	7.29
11	KAG1361275.1	56.38	catalase isozyme 2 (*Cocos nucifera*)	3	8.81
12	CL6681.Contig1_coconut	29.17	L-ascorbate peroxidase, cytosolic (*Elaeis guineensis*)	1	3.37
13	CL5565.Contig2_coconut	32.61	superoxide dismutase [Mn], mitochondrial (*Elaeis guineensis*)	3	11.78
14	KAG1330537.1	16.58	microsomal glutathione S-transferase 3 (*Cocos nucifera*)	2	11.49
15	CL45.Contig6_coconut	41.20	glutathione reductase, cytosolic (*Phoenix dactylifera*)	2	7.37
16	KAG1369736.1	29.80	2-Cys peroxiredoxin BAS1, chloroplastic (*Cocos nucifera*)	2	9.26
17	CL3017.Contig2_coconut	24.28	1-Cys peroxiredoxin (*Elaeis guineensis*)	8	40.83

N: Identification Numbers, FDR: False Discovery Rate, Uni. Pep.: Unique Peptides, Cov.: Protein coverage.

**Table 3:** Selected potential bioactive peptides of the globulin predicted by in-silico digestions with pepsin.

N	Proteins	Peptides	Modifications	PeptideRanker Score	Toxin Prediction
3	KAG1361520.1	QSFQQSESEQQGEKGQRRRSRDEHQRI	Gln->pyro-Glu (N-term Q)	0.870457	Non-Toxin
7	CL4719.Contig2_coconut	SEGWSRPF		0.853573	Non-Toxin
9	KAG1368674.1	QPVATPGMF	Gln->pyro-Glu (N-term Q) Oxidation (M)	0.851509	Non-Toxin
8	CL4719.Contig3_coconut	HPVATPGMF	Oxidation (M)	0.840543	Non-Toxin
7	CL4719.Contig2_coconut	HPVATPGMF	Oxidation (M)	0.840543	Non-Toxin
6	Unigene39216_coconut	MPGCPTTF	Oxidation (M)	0.83791	Non-Toxin
3	KAG1361520.1	QFGRSPW	Gln->pyro-Glu (N-term Q)	0.804836	Non-Toxin

N: Identification Numbers were according to Table 1.

**Table 4:** Selected potential bioactive peptides of the globulin predicted by in-silico digestions with trypsin.

N	Proteins	Peptides	Modifications	PeptideRanker Score	Toxin Prediction
5	CL3433.Contig1_coconut	EMGAMGDRTAVMLLMLLLSSWCLTAVTGNR	Oxidation (M)	0.998699	Non-Toxin
6	Unigene39216_coconut	SSTASLLSFSLCLLLLCHSSLSQRECQLDR		0.9969	Non-Toxin
3	KAG1361520.1	AMATSAATLLPFSLCLLLLCRASLAQFGR	Oxidation (M)	0.994361	Non-Toxin
3	KAG1361520.1	MKAMATSAATLLPFSLCLLLLCR	Met-loss+Acetyl (Protein N-term) Oxidation (M)	0.99011	Non-Toxin
7	CL4719.Contig2_coconut	AFVPFLLLLSILLVSATLTFSVTTEDPKR		0.98242	Non-Toxin
8	CL4719.Contig3_coconut	AFVPFLLLLSILLVSATLTFSVTTEDPKR		0.98242	Non-Toxin
2	CL8941.Contig1_coconut	MGKMASTLLATIYLWSLVAINGK	Oxidation (M)	0.977959	Non-Toxin
1	ALQ56981.1	EMDSDDDDEAEQEEDDVWTWRCLLK	Oxidation (M)	0.941081	Non-Toxin
7	CL4719.Contig2_coconut	MTTKPRAFVPFLLLLSILLVSATLTFSVTTEDPK	Acetyl (Protein N-term) Oxidation (M)	0.930602	Non-Toxin
8	CL4719.Contig3_coconut	MTTKPRAFVPFLLLLSILLVSATLTFSVTTEDPK	Acetyl (Protein N-term) Oxidation (M)	0.930602	Non-Toxin
7	CL4719.Contig2_coconut	MTTKPRAFVPFLLLLSILLVSATLTFSVTTEDPK	Met-loss+Acetyl (Protein N-term)	0.930602	Non-Toxin
8	CL4719.Contig3_coconut	MTTKPRAFVPFLLLLSILLVSATLTFSVTTEDPK	Met-loss+Acetyl (Protein N-term)	0.930602	Non-Toxin
9	KAG1368674.1	AYIPFLLLLSILFLSATLALSSNEQEDPELKQCK		0.927218	Non-Toxin
6	Unigene39216_coconut	QLMATSRSSTASLLSFSLCLLLLCHSSLSQR	Oxidation (M)	0.916637	Non-Toxin
6	Unigene39216_coconut	QLMATSRSSTASLLSFSLCLLLLCHSSLSQR	Gln->pyro-Glu (N-term Q)	0.916637	Non-Toxin
8	CL4719.Contig3_coconut	SWPFGESRRPFNLFHK		0.899487	Non-Toxin
1	ALQ56981.1	TASAILALLLLSSWSLMVVMAYQGRGMEGR	Oxidation (M)	0.879123	Non-Toxin
2	CL8941.Contig1_coconut	MASTLLATIYLWSLVAINGKDFPSFGPLVTR	Oxidation (M)	0.878424	Non-Toxin
7	CL4719.Contig2_coconut	DGPLELFAF		0.868349	Non-Toxin
2	CL8941.Contig1_coconut	SHPDPMR	Oxidation (M)	0.828137	Non-Toxin
1	ALQ56981.1	NRPQFLVGKSSLLHSMR	Oxidation (M)	0.823569	Non-Toxin
9	KAG1368674.1	QQPFYDEGMRR	Oxidation (M)	0.823569	Non-Toxin
9	KAG1368674.1	QQPFYDEGMRR	Gln->pyro-Glu (N-term Q)	0.815333	Non-Toxin
7	CL4719.Contig2_coconut	EGDPYFFDK		0.815321	Non-Toxin
9	KAG1368674.1	EGDPYFFDK		0.815321	Non-Toxin

N: Identification Numbers were according to Table 1.

**Table 5:** Selected potential bioactive peptides of the antioxidant proteins predicted by in-silico digestions with pepsin.

N	Proteins	Peptides	Modifications	PeptideRanker Score	Toxin Prediction
15	CL45.Contig6_coconut	SSEVVGGVGGTCVIRGCVPKKI		0.988623	Non-Toxin
2	CL2795.Contig1_coconut	APPVCCRF		0.969325	Toxin
10	KAG1355350.1	SGGSPFPGL		0.949596	Non-Toxin
1	CL7558.Contig1_coconut	PSGFPKSPF		0.893866	Non-Toxin
16	KAG1369736.1	FHPPMVSF	Oxidation (M)	0.888879	Toxin
1	CL7558.Contig1_coconut	SSKFPSGF		0.872751	Non-Toxin
2	CL2795.Contig1_coconut	RGVSFPF		0.866817	Non-Toxin
13	CL5565.Contig2_coconut	QGSGWVW	Gln->pyro-Glu (N-term Q)	0.84448	Non-Toxin
17	CL3017.Contig2_coconut	GISCDDVKCHMEW	Oxidation (M)	0.820966	Non-Toxin
10	KAG1355350.1	VGNNFPVF		0.818058	Non-Toxin
13	CL5565.Contig2_coconut	WKVMNW	Oxidation (M)	0.812069	Non-Toxin
9	KAG1327021.1	MSDWRTRPF	Oxidation (M)	0.801392	Non-Toxin

N: Identification Numbers were according to Table 2.

**Table 6:** Selected potential bioactive peptides of the antioxidant proteins predicted by in-silico digestions with trypsin.

N	Proteins	Peptides	Modifications	PeptideRanker Score	Toxin Prediction
14	KAG1330537.1	LKIGGFNFLALFGLIICTASSGIHLLIR		0.968709	Non-Toxin
6	CL1212.Contig1_coconut	FVTFCELLICFQWFHFAIGPTTVKVR		0.960217	Non-Toxin
1	CL7558.Contig1_coconut	FPSGFPKSPFR		0.954895	Non-Toxin
9	KAG1327021.1	HLQHSSALLAAVLAVVVALSFPAPSAAKLTPDYYQR		0.953075	Non-Toxin
14	KAG1330537.1	IGGFNFLALFGLIICTASSGIHLLIREVL		0.930888	Non-Toxin
6	CL1212.Contig1_coconut	GGHELSLTTGNAGGRLACGVVGLTPLE		0.92959	Non-Toxin
9	KAG1327021.1	FDNMYFK	Oxidation (M)	0.920339	Non-Toxin
11	KAG1361275.1	EGNWDLLGNNFPVFFIR		0.902904	Non-Toxin
16	KAG1369736.1	IFHPPMVSFLR	Oxidation (M)	0.890783	Non-Toxin
13	CL5565.Contig2_coconut	MPVAAFIFHCR	Oxidation (M)	0.8833	Non-Toxin
2	CL2795.Contig1_coconut	GVSFPFPVSSSSAAPPVCCR		0.882809	Toxin
12	CL6681.Contig1_coconut	NCAPLMLR	Oxidation (M)	0.877201	Non-Toxin
4	CL7860.Contig2_coconut	DLEILAFPCNQFLR		0.876087	Non-Toxin
9	KAG1327021.1	LFFHDCFVGGCDASILISSSAFNRAER		0.86701	Non-Toxin
8	Unigene14114_coconut	MQPSLSWPVVFLGLALLFFFLRNPTPPDK	Met-loss+Acetyl (Protein N-term)	0.855954	Non-Toxin
8	Unigene14114_coconut	MQPSLSWPVVFLGLALLFFFLRNPTPPDK	Acetyl (Protein N-term) Oxidation (M)	0.855954	Non-Toxin
1	CL7558.Contig1_coconut	SSAGGFLGDLIKWNFEK		0.851297	Non-Toxin
7	Unigene8126_coconut	NCAPIMLR	Oxidation (M)	0.850796	Non-Toxin
13	CL5565.Contig2_coconut	TLTLGLGFGPRGAVAASCPGLGSGLR		0.849617	Non-Toxin
11	KAG1361275.1	VHYVKFHWKPTCGVSCLLEEEATVVGGK		0.840276	Non-Toxin
9	KAG1327021.1	HLQHSSALLAAVLAVVVALSFPAPSAAK		0.836338	Non-Toxin
4	CL7860.Contig2_coconut	NKDLEILAFPCNQFLR		0.833597	Non-Toxin
1	CL7558.Contig1_coconut	SSLFQKNPSFAAKPLR		0.830042	Non-Toxin
2	CL2795.Contig1_coconut	ACSLLPPMLYSSPLALSR	Acetyl (Protein N-term) Oxidation (M)	0.826857	Non-Toxin
10	KAG1355350.1	EGNFDLVGNNFPVFFIR		0.826634	Non-Toxin
7	Unigene8126_coconut	TSTVLAQSAFGVAVAAGVVILSYLYEVSRR		0.82028	Non-Toxin
6	CL1212.Contig1_coconut	LDKFVTFCELLICFQWFHFAIGPTTVK		0.818727	Non-Toxin
16	KAG1369736.1	SFHGLRR		0.814079	Non-Toxin
10	KAG1355350.1	FHWRPTCGVKCLLEDEAVIVGGNNHSHATK		0.809949	Non-Toxin
9	KAG1327021.1	MARHLQHSSALLAAVLAVVVALSFPAPSAAK	Met-loss+Acetyl (Protein N-term)	0.80904	Non-Toxin
9	KAG1327021.1	MARHLQHSSALLAAVLAVVVALSFPAPSAAK	Acetyl (Protein N-term) Oxidation (M)	0.80904	Non-Toxin

N: Identification Numbers were according to Table 2.

## Data Availability

The data presented in this study are available on request from the corresponding author.
